# Pathophysiological functions of semaphorins in the sympathetic nervous system

**DOI:** 10.1186/s41232-023-00281-7

**Published:** 2023-06-08

**Authors:** Yumiko Mizuno, Yoshimitsu Nakanishi, Atsushi Kumanogoh

**Affiliations:** 1 Department of Respiratory Medicine and Clinical Immunology, Graduate School of Medicine, Osaka University, Suita, Osaka Japan; 2 Department of Immunopathology, World Premier International Research Center Initiative Immunology Frontier Research Center (WPI-IFReC), Osaka University, Suita, Osaka Japan; 3 Department of Advanced Clinical and Translational Immunology, Graduate School of Medicine, Osaka University, Suita, Osaka Japan; 4 Integrated Frontier Research for Medical Science Division, Institute for Open and Transdisciplinary Research Initiatives (OTRI), Osaka University, Suita, Osaka Japan; 5 Center for Infectious Diseases for Education and Research (CiDER), Osaka University, Suita, Osaka Japan; 6 Japan Agency for Medical Research and Development — Core Research for Evolutional Science and Technology (AMED–CREST), Osaka University, Suita, Osaka Japan; 7 Center for Advanced Modalities and DDS (CAMaD), Osaka University, Suita, Osaka Japan

**Keywords:** Sympathetic nervous system, Immunity, Inflammation, Semaphorins

## Abstract

Upon exposure to external stressors, the body senses them and activates the sympathetic nervous system (SNS) to maintain the homeostasis, which is known as the “fight-or-flight” response. Recent studies have revealed that the SNS also plays pivotal roles in regulating immune responses, such as hematopoiesis, leukocyte mobilization, and inflammation. Indeed, overactivation of the SNS causes many inflammatory diseases, including cardiovascular diseases, metabolic disorders, and autoimmune diseases. However, the molecular basis essential for SNS-mediated immune regulation is not completely understood. In this review, we focus on axon guidance cues, semaphorins, which play multifaceted roles in neural and immune systems. We summarize the functions of semaphorins in the crosstalk between the SNS and the immune system, exploring its pathophysiological roles.

## Background

The sympathetic nervous system (SNS) plays a crucial role in adapting to both external and internal stimuli. The SNS maintains the tissue homeostasis through modulating various biological processes including immune responses. Although recent studies have revealed the coupling of the SNS and the immune system, the molecular machinery of this coupling is largely unknown.

Semaphorins, initially identified as repulsive guidance factors [1, 2], are a family of secreted and membrane-bound molecules characterized by an extracellular N-terminal sema domain. In vertebrates, semaphorins are divided into five subfamilies from classes 3–7 and primarily exert their functions through plexins and neuropilins. Semaphorins play important roles in neural crest cell migration, ganglion formation, and axon guidance, which are essential for development of the SNS. In addition, some classes of semaphorins regulate immune responses, such as migration, antigen presentation, activation, proliferation, and antibody production [3]. These diverse functions of semaphorins highlight their significance in coupling the neural and immune systems.

In this review, we summarize the functions of semaphorins in the formation and maintenance of the SNS. In addition, we discuss the contribution of semaphorin signaling to SNS-mediated biological responses, particularly in the heart and brown adipose tissue (BAT), which have abundant sympathetic innervation.

### Physiology of the sympathetic nervous system

The SNS and parasympathetic nervous system (PNS) constitute the autonomic nervous system. The autonomic nervous system activates involuntary responses of peripheral organs. Specifically, the SNS is responsible for the “fight-or-flight” response by activating cardiovascular function, increasing muscle contractility, while suppressing gastrointestinal and urinary function. In contrast, the PNS is responsible for the rest and digestion response, decreasing heart rate, blood pressure, and muscle contractility while increasing gastrointestinal activity. Mammals respond to external and internal stressors via the two major stress-activated pathways, the hypothalamic–pituitary–adrenal (HPA) axis and the SNS [4]. Stress information is transmitted to the hypothalamus after passing through several intracerebral neural pathways. In the hypothalamus, corticotropin-releasing hormone (CRH)-containing neurons in the paraventricular nucleus (PVN) integrate stress information. These neurons make projections to the locus coeruleus of the brainstem, which in turn sends direct projections to sympathetic and parasympathetic preganglionic neurons. In principle, sympathetic postganglionic fibers release norepinephrine (NE), and parasympathetic postganglionic fibers release acetylcholine (Ach). Sympathetic activation also controls catecholamine biosynthesis and secretion from the adrenal medulla. Acute stressors, such as an imminent danger, hunger, sickness, and pain, elicit systemic SNS activation within seconds. The released catecholamine binds to membrane-bound G protein-coupled receptors, which initiate intracellular cyclic adenosine monophosphate signaling and activate cellular responses. This leads to the activation of the cardiovascular system and increased blood flow to vital organs [4].

In addition to the well-known functions described above, the SNS plays a vital role in maintaining tissue homeostasis by modulating the immune and metabolic system. Sympathetic nerve fibers in the bone marrow regulate the maintenance and retention of hematopoietic stem and progenitor cells via adrenergic receptors expressed on mesenchymal cells [5]. β-adrenergic signals in the bone marrow protect the niche from chemotherapy-induced insults [6]. In brown and beige adipose tissue, β-adrenergic signals mediate non-shivering thermogenesis and prevent obesity progression [7]. In the pancreas, β2-adrenergic signaling suppresses excess vasculature development and maintains insulin production [8]. In the skin, hyperactivation of SNS drives stress-induced gray hair by depleting melanocyte stem cells in hair follicles [9]. Acute stress induces sympathetic outflow to the β3-adrenergic receptors in BAT, which produces stress-induced interleukin-6 (IL-6) and promotes gluconeogenesis in the liver [10]. Thus, maintaining the SNS is critical for various pathophysiological processes.

### The sympathetic nervous system in immune regulation

In recent years, the crosstalk between the immune system and the nervous system, particularly the SNS, has been extensively studied and has gained attention. Sympathetic nerves are wired to primary and secondary lymphoid tissues, including bone marrow, lymph nodes, and the spleen. Lymphoid organs are highly innervated by adrenergic nerves, but only slightly by cholinergic nerves, indicating that the SNS is closely associated with immune functions of lymphoid organs [11, 12]. Among the adrenergic receptors, the β2-adrenergic receptor is the most abundantly expressed receptor on lymphocytes [13].

Catecholamines exert both pro-inflammatory and anti-inflammatory effects in a context-dependent manner, for example, depending on adrenoceptor subtype and the timing of adrenergic receptor activation. Both in vivo and in vitro studies have revealed that adrenergic signaling enhances or suppresses a variety of innate and acquired immune responses, including antigen presentation, cell proliferation, activation, trafficking, and antibody production [14, 15]. Adrenergic signaling primarily exerts immunosuppressive effects via β2 adrenergic signaling. For example, via β2-adrenergic signaling, NE suppresses antigen cross-presentation by dendritic cells [16], although NE promotes antigen presentation by B cells and antibody production [17]. NE also induces CD4^+^ T cell skewing toward Th17 over Th1 by suppressing interleukin-12p70 secretion from dendritic cells upon lipopolysaccharide challenge [18]. NE suppresses effector CD8^+^ T-cell function [19] while promotes inflammatory responses of memory CD8^+^ T cells [20]. Furthermore, NE downregulates natural killer cell activation in response to viral infection [21, 22]. Additionally, NE suppresses inflammatory cytokine production from macrophages [23] and group 2 innate lymphoid cell (ILC2) proliferation and effector function during parasitic infection [24].

Sympathetic activity affects peripheral leukocyte distribution in accordance with circadian rhythms. In mice, increased sympathetic input upregulates cell adhesion molecule expression on endothelial cells through β2- and β3-adrenergic receptors and recruits leukocytes to peripheral tissues at night [25]. NE induces lymphocyte retention in lymph nodes via activation of β2-adrenergic receptors on lymphocytes [26]. Lymphocytes are accumulated in the lymph nodes during sympathetic activation, and immunization during this period enhances the antibody response [27]. These studies suggest that adrenergic signaling influences the magnitude of the adaptive immune response by controlling immune cell distribution.

The SNS regulates the immune system in a direct or indirect manner, by acting on immune cells or on nonimmune cells, respectively. In white adipose tissue (WAT), the leptin–NE–β-adrenergic receptor axis induces lipolysis in adipocytes [28]. Adipose tissue macrophages (ATMs) display heterogenous responses to catecholamines. Some subsets of ATMs promote lipolysis and protect against obesity in response to SNS activation [29], while other subsets import and metabolize NE, thereby promoting obesity [30]. Perivascular adipocytes store catecholamine in response to NE signaling, leading to vasorelaxation [31]. Furthermore, some subsets of NE-stimulated macrophages release Ach, which in turn activates thermogenic adipocytes [32]. Sympathetic nerve fibers interact with β2-adrenergic receptors on mesenchymal stromal cells to maintain ILC2, a key player in type 2 immunity [33].

Recent technical advances in neurosciences, such as optogenetics, have facilitated the identification and manipulation of neural circuits that are essential for peripheral immune responses. Stressful situations, such as elevated platform stress, stimulate CRH-producing neurons in the PVN and the central amygdala, which are connected to the splenic nerve, leading to enhanced plasma cell formation and antigen-specific antibody response [34]. During acute restraint stress, leukocyte distribution is regulated by two distinct brain regions: the PVN and the motor cortex. CRH neurons in the PVN stimulate the HPA axis. Released glucocorticoids act directly on lymphocytes, inducing their homing to the bone marrow and reducing their sensitization at lymph nodes, which impairs host adaptive immunity to infection. The motor cortex–brain stem–skeletal muscle pathway mobilizes neutrophils to the site of injury via neutrophil-attracting C–X–C motif chemokine ligand 1, presumably participating in inflammation [35]. However, it remains to be elucidated how the neural circuits essential for immune responses are formed and maintained. Further investigation is required to reveal the entire regulatory machinery of neuro-immune coupling.

### The sympathetic nervous system in inflammatory diseases

Altered SNS signaling is associated with many pathological conditions. In autoimmune diseases, catecholamines modulate disease states in a phase-dependent manner. For example, in an experimental autoimmune encephalomyelitis (EAE) model, NE acts differently in the central and peripheral nervous systems at the induction and effector phases [36]. In the induction phase, β2-adrenergic signaling promotes microglial inflammation at spinal cord while suppresses CD4^+^ T-cell proliferation and skewing toward Th17 at the draining lymph nodes. In the effector phase, α1-adrenergic signaling activates microglia, macrophages, and dendritic cells and suppresses regulatory T-cell responses at both sites [36]. Chronic cold-stress stimulation ameliorates neuroinflammation by suppressing antigen presentation by monocytes in the bone marrow via β3-adrenergic receptor signaling [37]. In an EAE mouse model, pathogenic T-cell entry into the central nervous system (CNS) is induced by neural activation. Gravity-induced activation of sensory nerves leads to SNS-mediated C–C motif chemokine ligand 20 and IL-6 production in endothelial cells of dorsal blood vessel in the fifth lumbar cord, resulting in the accumulation of pathogenic CD4^+^ T cells in the CNS [38].

SNS dysfunction has been implicated in the development of cardiovascular diseases [39] and metabolic disorders [40]. SNS overactivation contributes to hypertension partly by stimulating the renin–angiotensin–aldosterone system [41]. Patients with type 2 diabetes show increased peak mean arterial pressure and blood pressure excursions throughout the day, which is partly due to the augmented sympathetic vascular transduction [42]. In diabetes, sympathetic nerve activation and catecholamine-producing leukocytes induces myelopoiesis in the spleen and accelerates atherosclerosis [43]. Excessive cardiac sympathetic activation in patients with chronic heart failure can lead to poor prognosis [44]. Inflammation in the stellate ganglia (STG), from which the postganglionic sympathetic neurons projecting to the heart primarily originate, is observed in patients with ventricular arrhythmias [45]. Macrophage depletion in the STG decreases cardiac sympathoexcitation and ventricular arrhythmias in rats with chronic heart failure [46]. These studies highlight the importance of regulating the sympathetic nerve activity for the treatment of cardiovascular and metabolic diseases. The crosstalk between the SNS and the immune system has attracted much attention, but the precise mechanisms of its regulation remain unclear.

### Semaphorins

Here, we focus on semaphorins as key molecules linking the nervous system and the immune system. Semaphorin family consists of over twenty members, which are divided into eight subclasses. Semaphorins are characterized by a common extracellular domain named the sema domain. Types 3–7 semaphorins are expressed in vertebrates. Type 3 semaphorins are secreted, whereas types 4–7 semaphorins are membrane-bound molecules. Semaphorins exert their functions via various receptors, including plexins and neuropilins. Semaphorins, originally identified as axonal guidance cues in the nervous system [1, 2], are essential for axon migration, growth, guidance, and neural regeneration in both the central and peripheral nervous systems. Additionally, semaphorins play pivotal roles in other physiological processes, such as angiogenesis [47], cellular morphology [48], bone homeostasis [49], and immune regulation [3, 50]. Semaphorins, plexins, and neuropilins expressed on immune cells regulate various immune responses including migration, proliferation, differentiation, and activation [3]. Altered semaphorin signaling is implicated in the pathogenesis of chronic inflammatory disorders, including autoimmune diseases [51], cancer [52], immunometabolic diseases [53], and cardiovascular diseases [54]. These studies highlight the diverse role of semaphorins in the neural and immune systems both in physiological and pathological conditions.

#### Semaphorin signaling in the development of the sympathetic nervous system

**Fig. 1:**
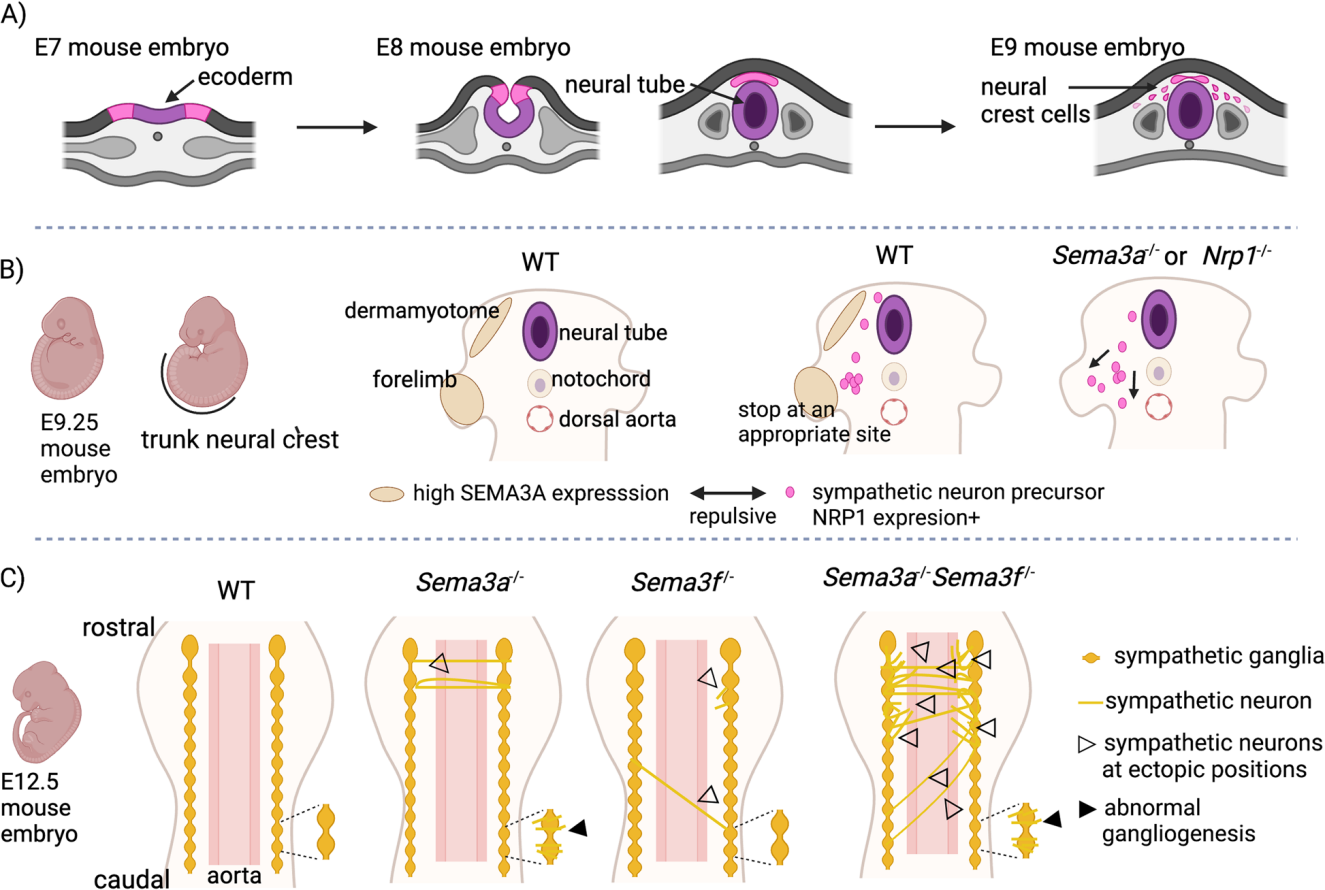
Semaphorin signaling in the SNS development. **A** Neural crest cells, differentiated from the ectoderm, migrate and aggregate around the developing aorta. **B** The SEMA3A–NRP1 signaling axis regulates the migration of sympathetic neuron precursors to the appropriate position. **C** SEMA3A and SEMA3F synergistically act in sympathetic nervous system patterning, such as sympathetic gangliogenesis and axon guidance. This image was created with BioRender (https://biorender.com/)

During embryonic development, the ectoderm differentiates into neural crest cells, which migrate along defined paths and differentiate into diverse cell types, including postganglionic sympathetic neurons [55] (Fig. 1A). Neural crest cells migrate ventrally and aggregate near the developing aorta, forming the sympathetic anlagen. Subsequently, sympathetic precursors then differentiate into neurons and segregate into the sympathetic trunk. Young sympathetic neurons send their axons to their appropriate targets to function properly [56, 57].

Semaphorin 3F (SEMA3F)–neuropilin 2 (NRP2) signaling is required for segmental neural crest migration. The repulsive interaction between NRP2 expressed on neural crest cells and SEMA3F on the posterior half of the somite restricts neural crest migration to the anterior half of each somite [58]. The SEMA3A–NRP1 signaling axis functions as a stop signal in the migration of the neural crest cells at an appropriate site. NRP1-expressing neural crest cells possibly stop following a SEMA3A concentration gradient. SEMA3A–NRP1 signaling aggregates sympathetic neuron precursors at defined target sites, leading to the establishment of axon fasciculation [59] (Fig. 1B). SEMA3A–NRP1 and SEMA3F–NRP2 signaling synergistically tune sympathetic gangliogenesis, neurite guidance, and neural projection to the target organ. Mutants lacking both SEMA3A and SEMA3F or both NRP1 and NRP2 exhibited more ectopic ganglionic neurites and ectopic axons extending across the midline, compared to the single mutants [60] (Fig. 1C). Plexin-A3 (PLXNA3) and PLXNA4 signaling also regulate sympathetic neural guidance and the formation of the ganglia such as the superior cervical ganglia, the STG, and sympathetic chain ganglia. Semaphorin signaling through PLXNA3 and PLXNA4 restricts the migration of sympathetic neurons redundantly. Ectopic axons and neurons are observed only in double mutant mice [57]. SEMA3A, PLXNA3, and NRP1 play crucial roles in the maintenance of sympathetic ganglia by inducing cell death in immature neurons [61]. When SEMA3A binds to neuronal growth cones, the SEMA3A and NRP1–PLXNA3 receptor complex is endocytosed at the axon terminal and retrogradely transported to cell bodies, inducing neuronal apoptosis. This phenomenon mainly occurs in immature neurons. Overall, semaphorin signaling plays critical roles in various steps of sympathetic development.

#### Semaphorin-mediated sympathetic control in the cardiovascular system

In addition to its roles in the development of the SNS, semaphorin signaling is also important for regulating sympathetic tones to target organs. The heart is highly innervated by autonomic nerves and is tightly regulated. Cardiac postganglionic sympathetic neurons, originated mainly from the STG, are primarily located in the subepicardium of the ventricle. Altered sympathetic innervation is observed in patients with atrial fibrillation and heart failure [62]. Semaphorin signaling contributes to heart development mainly in vascular patterning and cardiac morphogenesis [63], and dysregulation of semaphorin signaling has been implicated in several cardiovascular diseases [54].

Here, we highlight the roles of semaphorin in regulating the SNS in the cardiovascular system both physiologically and pathologically. Various semaphorin signals, such as SEMA3s, SEMA4s, and SEMA6s, play important roles in the regulation of angiogenesis, endothelial cell migration, and lymphangiogenesis in cardiovascular development [63]. Among them, SEMA3A is known as a direct regulator of the SNS innervation in the heart and is associated with cardiovascular disease.

**Fig. 2:**
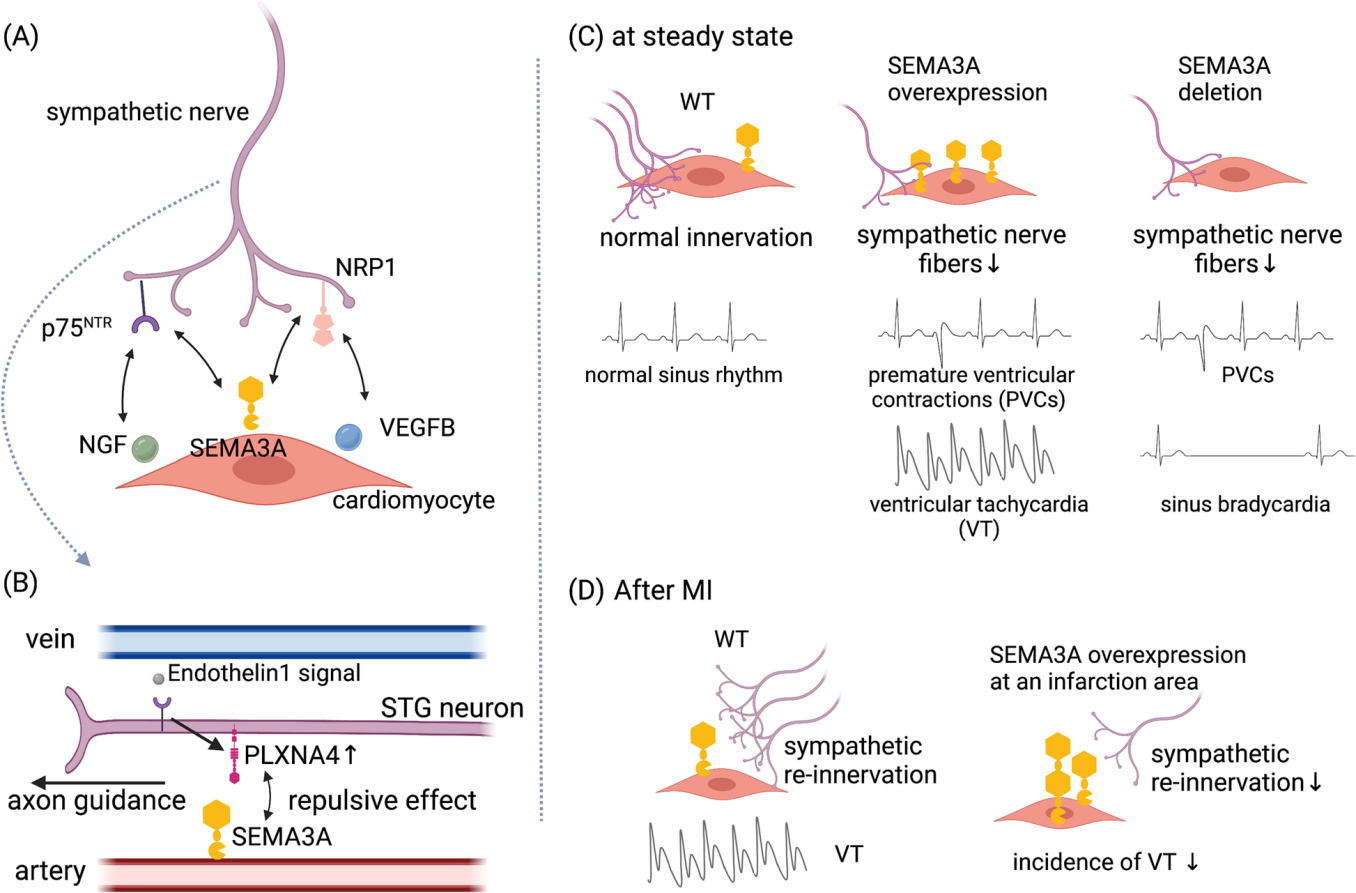
Cardiac sympathetic regulation by semaphorins. **A** Semaphorin signaling regulates cardiac innervation in combination with other neural cues and growth factors. **B** The SEMA3A–PLXNA4 repulsive interaction controls axon trajectory of the STG neurons. **C** SEMA3A maintains normal cardiac sympathetic innervation and functions. Both overexpression and deletion of SEMA3A induce abnormal sympathetic nerve innervation and lethal arrhythmias. **D** After MI, SEMA3A protects against ventricular arrhythmias by reducing sympathetic hyper-reinnervation. This image was created with BioRender (https://biorender.com/)

Cardiac sympathetic nerves receive both nerve growth factor (NGF) and SEMA3A signaling. NGF is a chemoattractant, and SEMA3A is a chemorepellent for sympathetic neurons. Sympathetic neurons lacking p75 neurotrophin receptor (p75^NTR^), which is one of NGF receptors, show a high sensitivity to SEMA3A in axon growth [64]. Overexpression of vascular endothelial growth factor-β (VEGF-β) in myocardium, which competes with SEMA3A for binding to NRP1, promotes ventricular arrhythmias due to enhanced sympathetic nerve sprouting in the myocardium [65]. These results suggest that semaphorins and their receptors regulate cardiac innervation properly in combination with other neural guidance cues and growth factors (Fig. 2A). The SEMA3A–PLXNA4 signaling is involved in guiding cardiac sympathetic axons. Venous endothelin initiates axonal extension toward the vein, and endothelin-1 induces PLXNA4 expression in the STG neurons via myocyte enhancer factor 2c. PLXNA4 expressed on the STG neurons serves as a repulsive guidance receptor for arterial SEMA3A, ensuring that axons extend along venous routes [66] (Fig. 2B).

Genetic variants of *SEMA3A* are associated with idiopathic ventricular fibrillation in humans. Patients with *SEMA3A* mutations exhibit an abnormal elongation of the cardiac sympathetic nerve fibers with diminished repellent activities mediated by *SEMA3A* mutations [67]. SEMA3A properly regulates sympathetic cardiac innervation and prevents sudden death from arrhythmia [68]. In the developing heart, Purkinje fibers synthesize SEMA3A. Most *Sema3a*^*−/−*^ mice die of sinus bradycardia and premature ventricular contractions due to disrupted cardiac sympathetic innervation patterning within the first postnatal week. Cardiac-specific overexpression of SEMA3A increases the risk of death from ventricular arrhythmia due to a marked decrease in intramyocardial cardiac sympathetic nerve fibers, increased catecholamine sensitivity, and prolonged myocardial action potential duration (Fig. 2C). Furthermore, SEMA3A exerts protective effects in myocardial infarction (MI). Overexpression of SEMA3A in the myocardium reduces ventricular arrhythmias by downregulating sympathetic reinnervation in the rat MI model [69] (Fig. 2D). Given that supplementation of SEMA3A improves cardiac autonomic disorders after MI in rodents, SEMA3A may be a potential therapeutic target of cardiovascular diseases in human [70]. Collectively, SEMA3A regulates cardiac performance via modulation of sympathetic nerve distribution and function.

### Sympathetic control of BAT function mediated by semaphorins

**Fig. 3:**
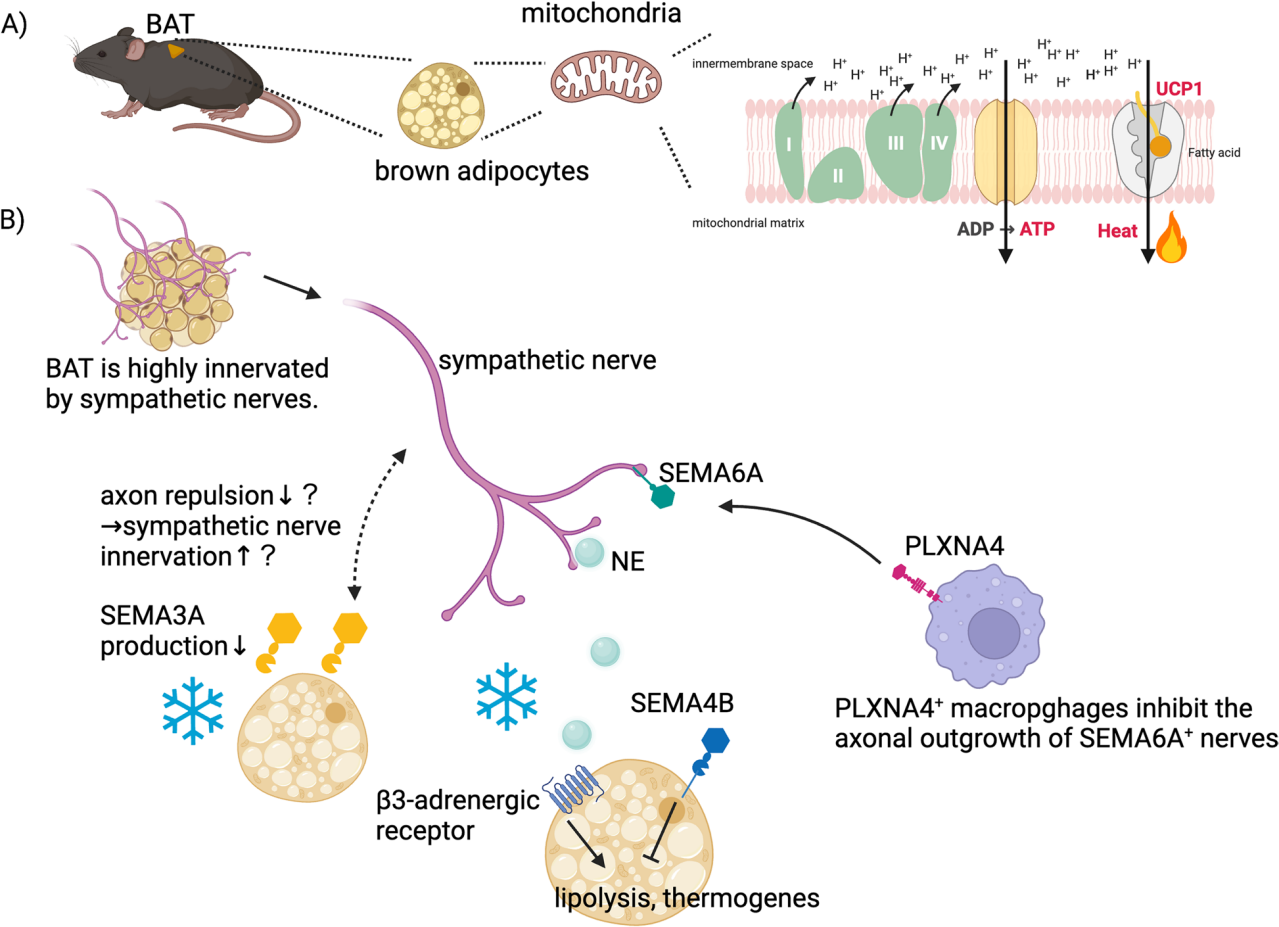
Semaphorin signaling in SNS-mediated BAT functions. **A** Brown adipocytes generate non-shivering thermogenesis via the UCP1 uncoupling system, which can use the electrochemical gradient of protons across the inner mitochondrial membrane to generate heat instead of adenosine triphosphate. **B** Semaphorin signaling modulates BAT activity via SNS. PLXNA4-expressing macrophages inhibit axonal outgrowth of SEMA6A-expressing sympathetic nerves. Cold exposure suppresses SEMA3A production, possibly affecting axon repulsion. SEMA4B protects against excessive lipolysis mediated by β3-adrenergic signaling. This image was created with BioRender (https://biorender.com/)

BAT is highly innervated by sympathetic nerves and is the main site of adaptive thermogenesis. In rodents, the intrascapular region contains the largest BAT depots, while in humans, they are mainly located in the supraclavicular, paraspinal, and axillary regions. Postganglionic sympathetic neurons projecting to intrascapular BAT originate from stellate/T1 ganglion and T2–T5 sympathetic chain ganglia in mice [71]. Brown adipocytes generate heat by a specific uncoupling mechanism via the uncoupling protein-1 (UCP1) in mitochondria [72] (Fig. 3A). NE released from sympathetic nerve terminals mediates thermogenesis via β2- and β3-adrenergic receptors in adipocytes [72, 73]. In addition, BAT secretes several endocrine molecules that interact with other organs, such as the bone, brain, and liver [10, 74]. The activation of BAT function is a potential therapeutic strategy for cardiometabolic diseases in both mice and humans [75].

Several reports suggest that semaphorins and their receptors play important roles in the maintenance of BAT function (Fig. 3C). PLXNA4 expressed on macrophages interacts with SEMA6A expressed on sympathetic nerve fibers to inhibit innervation into BAT, leading to fat accumulation in brown adipocytes and impaired steady-state energy expenditure at thermoneutrality [29]. NRP1-expressing myeloid cells in WAT protect against metabolic syndrome by supporting vasculature formation and maintaining the metabolic capacity [76]. NRP1 is also highly expressed in BAT-resident macrophages. NRP1-expressing myeloid cells maintain regular vascular morphology and sympathetic nerve fiber innervation in the BAT, which leads to the prevention of high-fat diet-induced BAT hypertrophy and the maintenance of core body temperature during cold exposure [77]. Some studies have also implicated the roles of SEMA4B in BAT. Insulin stimulation induces phosphorylation of SEMA4B in BAT, but not in other tissues [78], suggesting that phosphorylated SEMA4B signaling is important for BAT-specific functions such as non-shivering thermogenesis. SEMA4B is known as a novel adipokine, which is cleaved by a disintegrin and metalloprotease 17 (ADAM17) [79]. The SEMA4B–ADAM17 signaling axis suppresses excessive energy expenditure downstream of NE signaling. NE triggers ADAM17-induced cleavage of SEMA4B. Cleaved SEMA4B interacts with plexins on brown adipocytes and suppresses adipocyte differentiation, lipolysis, and thermogenesis. Furthermore, cold stimulation suppresses SEMA3A secretion from brown adipocytes, possibly affecting SEMA3A-mediated axon repulsion [80]. Collectively, these findings indicate that the SNS and semaphorin signaling cooperatively maintain BAT homeostasis.

## Conclusions

The physiological functions of SNS have been extensively studied. Recent technological advancements have facilitated the manipulation of activity in specific neural populations in the brain, enabling the identification of specific neural circuits for each biological process. The communication between the SNS and immune system is well established, and semaphorins are believed to be the key molecules that link these systems. As discussed in this review, semaphorins regulate not only the development and function of the SNS but also immune responses. Thus, semaphorins could be promising therapeutic targets for SNS-related chronic inflammation. However, it remains unclear whether and how semaphorins exert organ- or cell-specific functions to couple sympathetic and immune responses. In summary, a comprehensive understanding of semaphorin signaling would be beneficial for regulating SNS-related pathologies including chronic inflammation.

## Data Availability

Not applicable.

## Abbreviations

SNS: Sympathetic nervous system
BAT: Brown adipose tissue
PNS: Parasympathetic nervous system
HPA: Hypothalamic-pituitary-adrenal
CRH: Corticotropin-releasing hormone
PVN: Paraventricular nucleus
NE: Norepinephrine
Ach: Acetylcholine
IL-6: Interleukin-6
ILC2: Group 2 innate lymphoid cell
WAT: White adipose tissue
ATMs: Adipose tissue macrophages
EAE: Experimental autoimmune encephalomyelitis
CNS: Central nervous system
STG: Stellate ganglia
SEMA: Semaphorin
NRP: Neuropilin
PLXN: Plexin
NGF: Nerve growth factor
p75^NTR^: p75 neurotrophin receptor
VEGF: Vascular endothelial growth factor
MI: Myocardial infarction
UCP1: Uncoupling protein-1
ADAM: A disintegrin and metalloprotease
